# Pannexin-1-dependent caspase-1 activation and secretion of IL-1β is regulated by zinc

**DOI:** 10.1002/eji.200838843

**Published:** 2009-02

**Authors:** David Brough, Pablo Pelegrin, Nancy J Rothwell

**Affiliations:** Faculty of Life Sciences, University of ManchesterManchester, UK

**Keywords:** Caspase-1, IL-1, Inflammation, Macrophage, Zinc

## Abstract

Inflammatory processes induced by IL-1β are critical for host defence responses, but are also implicated in disease. Zinc deficiency is a common consequence of, or contributor to, human inflammatory disease. However, the molecular mechanisms through which zinc contributes to inflammatory disease remain largely unknown. We report here that zinc metabolism regulates caspase-1 activation and IL-1β secretion. One of the endogenous mediators of IL-1β secretion is adenosine triphosphate, acting *via* the P2X7-receptor and caspase-1 activation in cells primed with an inflammatory stimulus such as LPS. We show that this process is selectively abolished by a brief pre-treatment with the zinc chelator *N,N,N′,N′*-tetrakis-(2-pyridylmethyl) ethylene diamine (TPEN). These effects on IL-1β secretion were independent of rapid changes in free zinc within the cell, not a direct effect on caspase-1 activity, and upstream of caspase-1 activation. TPEN did however inhibit the activity of pannexin-1, a hemi-channel critical for adenosine triphosphate and nigericin-induced IL-1β release. These data provide new insights into the mechanisms of caspase-1 activation and how zinc metabolism contributes to inflammatory mechanisms.

## Introduction

The pro-inflammatory cytokine IL-1β is a key mediator of host-defence responses, which also contributes to the pathogenesis of diverse diseases 1. Its expression and release are controlled at multiple levels by cells of the innate immune system and this currently constitutes an intense area of research. IL-1β is expressed in response to stimulation of pattern recognition receptors of the TLR family, as an inactive 31 kDa precursor pro-IL-1β 1, which is not released from intact cells. Pro-IL-1β is expressed without a signal sequence 2 and is translated in the cytoplasm of macrophages stimulated with bacterial endotoxin (LPS) 3. Cells expressing pro-IL-1β require a second stimulus to activate the protease caspase-1 that cleaves pro-IL-1β to IL-1β, which is then secreted.

A number of triggers for caspase-1 activation have been identified; all activate cytosolic pattern recognition receptors and induce the assembly of inflammasome complexes that result in the autocatalytic activation of caspase-1 4. Extracellular adenosine triphosphate (ATP) has been studied widely as a second stimulus and regulates the activation of caspase-1 through stimulation of the cell surface P2X7 receptor 5 and *via* assembly of a NACHT-, LRR-, and PYD-containing protein3 (NALP3) inflammasome 6.

Zinc is essential for immune system function, and zinc deficiency contributes to many clinical disorders 7. At the cellular level, zinc modulates a number of signalling mechanisms central to innate immunity 8,9. Zinc may act by regulating protein function directly through binding to a motif such as a zinc-finger 10, or alternatively increases in free or labile zinc may act as a second messenger 11.

In this study we tested the hypothesis that zinc contributes to the activation of caspase-1 and the subsequent secretion of IL-1β from primary mouse macrophages. Changes in the concentration of potassium, calcium and chloride are known to influence the ATP-induced processing and secretion of IL-1β acting upstream of caspase-1 12–14, and we now show that the activity of pannexin-1, a key regulatory element in the release of IL-1β, is zinc-dependent.

## Results and discussion

### Effects of zinc chelation on IL-1β secretion

**Figure 1:**
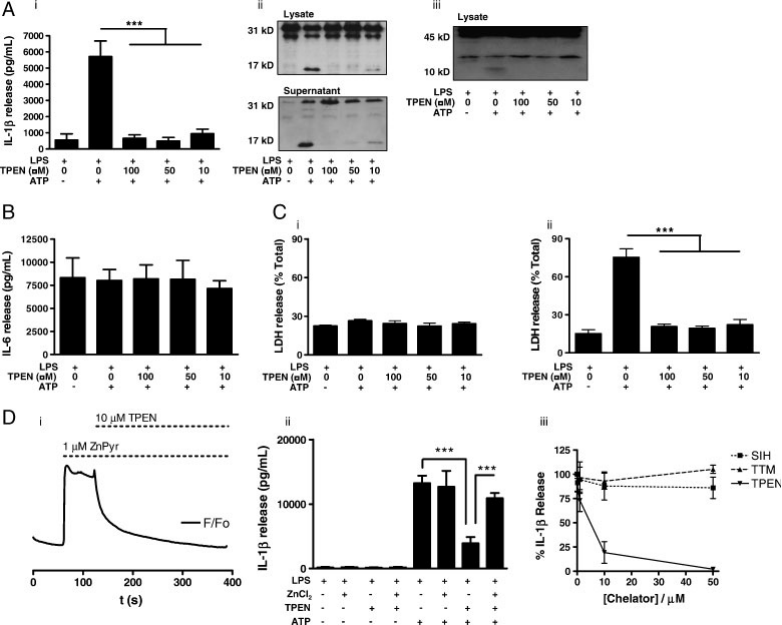
IL-1β secretion from LPS-treated mouse macrophages is zinc dependent. (A) Primary cultured mouse peritoneal macrophages were treated with LPS (1 μg/mL, 2 h) prior to incubation with vehicle (0.5% DMSO) or with the zinc chelator TPEN (10, 50 and 100 μM, 15 min). The inhibitory effects of zinc chelation on ATP-induced (5 mM, 10 min) IL-1β secretion were determined by ELISA (i). Pro-IL-1β processing was determined by Western blot; the 31 kDa band is pro-IL-1β and the 17 kDa band is the caspase-1 cleavage product; cleavage was inhibited by TPEN (ii). Caspase-1 activation was measured by cleavage of the 45 kDa pro-caspase-1 and detection of a 10 kDa sub-unit (iii), which was also inhibited after incubation with the zinc chelator TPEN. (B) The effects of TPEN and ATP on IL-6 release from the experiment described in (A) were determined by ELISA. (C) The effects of TPEN and ATP on cell death from the experiment described in (A) were determined by a LDH assay (i). The protective effects of TPEN on macrophage cell death induced by a 5 min pulse with 5 mM ATP followed by a 25 min incubation were measured by an LDH assay (ii). (D) Changes in FluoZin-3 fluorescence in response to 1 μM ZnPyr applied 1 min before the addition of 10 μM TPEN was determined using the BD Pathway Bioimager (i). Under the conditions described in (A), but with a media change prior to ATP stimulation, the inhibitory effects of TPEN (10 μM, 19 min) on IL-1β secretion can be overcome by coincubation with ZnCl_2_ (50 μM, 15 min) (ii). IL-1β release from LPS (1 μg/mL, 2 h) and ATP (5 mM, 10 min) treated peritoneal macrophages were not inhibited by the copper chelator TTM or the iron chelator SIH but was abolished by the zinc chelator TPEN (iii). Data show the mean±SD of at least three independent experiments. Blots are representative of three experiments. ^***^p<0.001.

We first tested the hypothesis that zinc contributes to caspase-1-dependent pro-IL-1β cleavage and mature IL-1β release by exposing LPS-primed (1 μg/mL, 2 h) mouse peritoneal macrophages to the zinc chelator *N,N,N′,N′*-tetrakis-(2-pyridylmethyl) ethylene diamine (TPEN) for 15 min prior to 10 min incubation with 5 mM ATP. Pre-treatment of LPS-primed mouse peritoneal macrophages with TPEN abolished the release of IL-1β in response to ATP (Fig. 1Ai). This was due to inhibition of pro-IL-1β processing (Fig. 1Aii) resulting from an inhibition of caspase-1 activation, as shown by the loss of the active p10 caspase-1 sub-unit (Fig. 1Aiii). These results indicate a previously unknown involvement of zinc in this key pro-inflammatory event. Importantly, under these conditions, the production of the pro-inflammatory cytokine IL-6 was not affected by TPEN, suggesting the specificity of the effect (Fig. 1B).

In the experiment described in the previous paragraph, macrophages were incubated with ATP for 10 min. We have reported recently that after this period of stimulation the macrophages have released significant quantities of IL-1β without leaking the cytosolic protein lactate dehydrogenase (LDH), which is used as a marker for a loss of membrane integrity 3. Under these conditions ATP did not increase LDH release above control levels and there was no effect of TPEN (Fig. 1Ci). However, stimulation of P2X7-receptor-induced IL-1β release from LPS-primed macrophages does result in macrophage cell death when assayed at later time points, and this is dependent on caspase-1 15. Thus, we hypothesised that TPEN would also block P2X7-receptor-induced cell death. We tested the effects of TPEN on the cell death induced by a 5 min ATP pulse, followed by 25 min incubation in the absence of ATP. We observed that a 15 min pre-incubation with TPEN completely inhibited ATP-induced cell death (Fig. 1Cii). These data suggest therefore that the mechanisms of ATP-induced and caspase-1-dependent cell death and IL-1β release are both regulated by zinc.

We next tested the hypothesis that the inhibitory effects of TPEN on IL-1β secretion would be overcome by the addition of excess zinc. To test this ZnCl_2_ was added to TPEN-containing macrophages. However, to ensure that the addition of ZnCl_2_ did not bind TPEN extracellularly, and prevent its cellular accumulation, we performed an experiment to demonstrate TPEN loading. LPS-primed macrophages were loaded with the selective Zn^2+^ indicator FluoZin-3 16 and imaged on a BD Pathway Bioimager. FluoZin-3-loaded macrophages were incubated with 1 μM of the Zn^2+^ ionophore, 1-hydroxypyridine-2-thione (zinc salt) (ZnPyr) for 1 min to increase the level of labile zinc (Fig. 1Di). TPEN (10 μM) was then added to the well and a rapid drop in cellular FluoZin-3 fluorescence was observed, returning to baseline within 4 min (Fig. 1Di). This demonstrated that within 4 min of TPEN loading there was sufficient within the cell to abolish a ZnPyr-induced increase in labile zinc. Therefore, ZnCl_2_ (50 μM) was added to the cells 4 min after the addition of TPEN and incubated for a further 15 min.

After 15 min the media were removed from all wells and fresh media or fresh media containing 5 mM ATP were added for a further 10 min. The supernatants were then analysed for released IL-1β by ELISA. The media change after the TPEN incubation was introduced since μM zinc is known to inhibit P2X7-receptor function 17 and may thus hinder interpretation of data. There was no difference in the levels of IL-1β released from LPS-primed macrophages when treated alone with vehicle, TPEN, ZnCl_2_ or TPEN plus ZnCl_2_ (Fig. 1Dii). ATP induced IL-1β release, and this was inhibited by TPEN (Fig. 1Dii). ZnCl_2_ had no effect on ATP-induced IL-1β release but did rescue the effects of TPEN (Fig. 1Dii). Thus, these data strongly suggest the zinc dependence of ATP-induced IL-1β release.

TPEN binds zinc preferentially, but also binds iron and copper, but not calcium or magnesium ions at biologically relevant levels 18. Therefore, we tested the effects of other selective metal ion chelators. The selective iron chelator, salicylaldehyde isonicotinoyl hydrazone (SIH), and the selective copper chelator, ammonium tetrathiomolybdate (TTM), had no effect on ATP-induced IL-1β secretion from LPS-primed macrophages (Fig. 1Diii), suggesting that the effect of TPEN was due to its binding of zinc.

### Zinc-dependent mechanism

**Figure 2:**
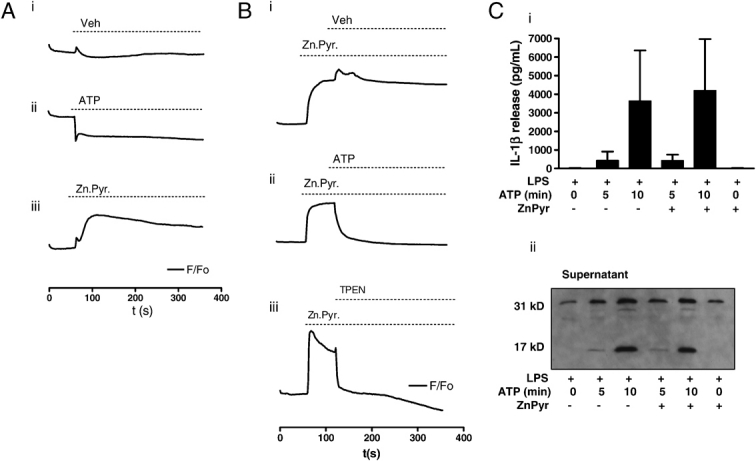
Rapid changes in labile zinc and effects on IL-1β secretion in mouse macrophages. (A) Primary cultured mouse peritoneal macrophages were treated with LPS (1 μg/mL, 2 h), and changes in FluoZin-3 fluorescence in response to vehicle (i), 5 mM ATP (ii) or 1 μM ZnPyr (iii) were determined using the BD Pathway Bioimager. (B) Under the conditions described in (A), FluoZin-3 fluorescence was used to record an increase in the labile zinc pool in response to 1 μM ZnPyr and how this was affected by the addition of vehicle (i), 5 mM ATP (ii) or 50 μM TPEN (iii). (C) Peritoneal macrophages were treated with LPS (1 μg/mL, 2 h)±1 min pre-incubation with 1 μM ZnPyr. The effects of a 5 and 10 min incubation with 5 mM ATP on IL-1β release was measured by ELISA (i), and pro-IL-1β processing was measured by Western blot (ii). ELISA results show the mean±SD of at least three independent experiments. Blots are representative of three experiments.

Increases in intracellular zinc ([Zn^2+^]_i_) and the Zn^2+^ wave (comparable to a Ca^2+^ wave) have been suggested recently to act as second messengers in immune cells 11. To examine whether this possibility could account for the effects of TPEN described in the previous section, we investigated the effect of P2X7-receptor activation on [Zn^2+^]_i_ and tested whether manipulation of this influenced IL-1β release. LPS-primed macrophages loaded with FluoZin-3 were imaged for 1 min prior to the addition of vehicle (Fig. 2Ai), 5 mM ATP (Fig. 2Aii) or 1 μM ZnPyr (Fig. 2Aiii). Addition of vehicle had no noticeable effect on [Zn^2+^]_i_ (Fig. 2Ai). In contrast, stimulation of the P2X7-receptor with 5 mM ATP induced a robust decline from baseline fluorescence (Fig. 2Aii). Incubation with the ionophore ZnPyr caused an increase in [Zn^2+^]_i_ (Fig. 2Aiii). The FluoZin-3 fluorescence baseline detected in the unstimulated cells likely represents the pool of labile zinc. Manipulation of the labile zinc pool by P2X7-receptor activation in this way may represent an efflux of zinc through the P2X7 channel.

We manipulated the labile zinc pool further to investigate how this affects IL-1β processing and release. LPS-primed macrophages, loaded with FluoZin-3, were imaged for 1 min, treated with ZnPyr (1 μM), then imaged for a further minute and subjected to addition of vehicle, ATP (5 mM) or TPEN (50 μM) to assess the effects on FluoZin-3 fluorescence. Addition of vehicle 1 min after the addition of ZnPyr had no effect on [Zn^2+^]_i_ (Fig. 2Bi). Addition of ATP again induced a rapid drop in [Zn^2+^]_i_ (Fig. 2Bii), confirming our earlier observation (Fig. 2Aii). TPEN, added 1 min after treatment with ZnPyr, resulted in a rapid drop in cellular fluorescence, confirming that we were indeed manipulating levels of labile zinc (Fig. 2Biii).

These imaging experiments indicate that the changes in [Zn^2+^]_i_ observed in response to ATP stimulation are unlikely to be important for the activation of caspase-1 because if a drop in [Zn^2+^]_i_ was important, the effects of ATP on IL-1β release would be similar to that of TPEN. Importantly, we did not observe a rise in FluoZin-3 fluorescence in response to ATP suggesting that we were not inducing a second messenger action of zinc as described previously 11. We also tested the hypothesis that a short (1 min) pre-incubation with ZnPyr would not inhibit ATP-induced processing and secretion of IL-1β. The incubation with ZnPyr would increase cellular labile zinc and buffer its loss after ATP stimulation. We have reported previously that secretion of IL-1β from murine macrophages is minimal after 5 min incubation with ATP (5 mM) but almost complete at 10 min 3. We measured IL-1β release 5 or 10 min after incubation with ATP (5 mM), with and without a 1 min pre-incubation with ZnPyr (1 μM). Secretion of IL-1β after 5 min incubation with ATP was less than that with a 10 min incubation and release at both times was completely unaffected by ZnPyr (Fig. 2Ci). Analysis of the supernatants confirmed that the processing of pro-IL-1β was also unaffected (Fig. 2Cii). The effect of a 10 min incubation with ZnPyr (1 μM) alone is also shown, confirming that IL-1β processing and release is independent of increases in [Zn^2+^]_i_ (Fig. 2C). Thus, these data suggest that rapid changes in labile zinc do not contribute to ATP-induced caspase-1 activation and IL-1β secretion.

**Figure 3:**
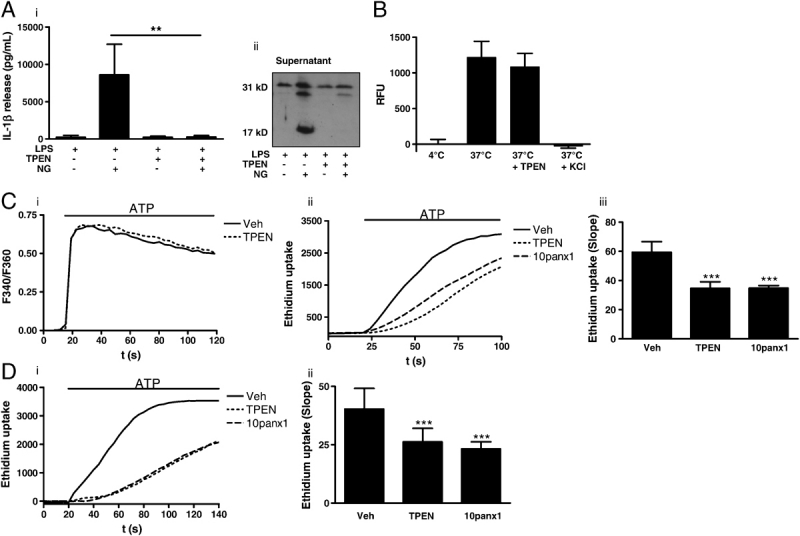
Zinc-dependent mechanism. (A) Nigericin (20 μM, 10 min)-induced IL-1β release (i) and pro-IL-1β processing (ii) in LPS-treated (1 μg/mL, 2 h) peritoneal macrophages was inhibited by TPEN. (B) *In vitro* inflammasome assembly and caspase-1 activity, induced by hypotonic lysis of LPS-treated peritoneal macrophages was measured by Ac-YVAD-AMC cleavage, and was not inhibited by TPEN. RFU, relative fluorescence units. (C) The effects of a 20 min incubation of 50 μM TPEN on the Fura-2 (F340/F360), [Ca^2+^]_i_ response to 1 mM ATP in P2X7 expressing HEK-293 cells (i). Representative fluorescence traces showing the effects of vehicle (DMSO), TPEN (50 μM), and ^10^panx1 (mimetic pannexin-1 peptide, 400 μM), on ATP (3 mM) induced ethidium dye uptake in P2X7 expressing HEK-293 cells (ii). A summary of the slope of ethidium dye uptake in P2X7 expressing HEK-293 cells (iii). (D) Representative fluorescence traces showing the effects of vehicle (DMSO), TPEN (50 μM), and ^10^panx1 (mimetic pannexin-1 peptide, 400 μM), on ATP (3 mM) induced ethidium dye uptake in LPS-treated primary mouse peritoneal macrophages (i). A summary of the slope of ethidium dye uptake in primary mouse peritoneal macrophages (ii). Data show the mean±SD of at least three independent experiments. Fluorescence traces are representative of at least three separate experiments. ^***^p<0.001, ^**^p<0.01.

It is possible that TPEN interferes directly with ATP binding to the P2X7-receptor. To address this possibility we investigated the effects of TPEN on IL-1β secretion induced by the K^+^ ionophore nigericin, which induces caspase-1-dependent secretion of IL-1β independent of the P2X7-receptor 15. Pre-treatment of LPS-primed macrophages with TPEN (50 μM, 15 min) abolished nigericin (20 μM, 10 min) induced secretion of IL-1β (Fig. 3Ai) and processing of pro-IL-1β (Fig. 3Aii), suggesting that TPEN acts independent of the P2X7-receptor.

The inflammasome constitutes the large protein complex required for the activation of caspase-1 19. Lysis of LPS-primed cells in a hypotonic solution containing little or no K^+^ enables oligomerisation of the adaptor proteins required to reconstitute a NALP inflammasome *in vitro* 19,20. Increasing K^+^ concentrations inhibits inflammasome assembly 20. We used this assay to test whether TPEN had any direct effects on inflammasome assembly, or caspase-1 activity. Cleavage of the fluorescent caspase-1 substrate Ac-YVAD-AMC was used as an indicator for inflammasome assembly. At 4°C no active inflammasomes were formed (Fig. 3B). However, incubation of lysates at 37°C induced a robust activation of caspase-1 activity that was inhibited by 130 mM/K^+^ as reported previously 20. Co-incubation with TPEN (50 μM) had no effect on caspase-1 activity induced in this assay (Fig. 3B). These data suggest that TPEN is not a direct inhibitor of inflammasome assembly or of caspase-1 activity.

Pannexin-1 is a membrane protein in mammalian cells, which is structurally related to invertebrate hemichannel proteins and is known to be essential for both ATP- and nigericin-induced caspase-1 activation and IL-1β secretion 21,22. We tested whether pannexin-1 activity was zinc-dependent. The nigericin data suggested that the zinc-dependent effect could be independent of P2X7-receptor activation (Fig. 3A). We confirmed that TPEN did not inhibit P2X7-receptor activation by measuring changes in [Ca^2+^]_i_ in response to 1 mM ATP in vehicle and TPEN (50 μM, 15 min) loaded human embryonic kidney (HEK)-293 cells over-expressing the mouse P2X7-receptor, and loaded with Fura-2 (Fig. 3Ci). Pannexin-1 is essential for a component of the dye uptake pathway induced by P2X7-receptor activation since RNAi knockdown, or inhibition by a mimetic peptide (^10^panx1) delays dye uptake rather than abolish it 22. We found that a short incubation (20 min) with TPEN (50 μM) inhibited ATP-induced ethidium dye uptake to the same extent as incubation with the pannexin-1 mimetic peptide ^10^panx1 in the P2X7 expressing HEK-293 cells (Fig. 3Cii and iii).

These data suggested that the zinc-dependent step was the activation of pannexin-1. P2X7-receptor over-expressing HEK-293 cells provide a robust model for investigating P2X7, and pannexin-1-dependent mechanisms 21,22 and we confirmed that the effect was also present in LPS-treated primary mouse peritoneal macrophages (Fig. 3Di and ii).

## Concluding remarks

In summary, the data presented in this manuscript provide evidence that zinc is an intrinsic regulator of caspase-1 activation and IL-1β secretion. This effect is independent of rapid changes in free zinc, suggesting that it is not exerting its effect as a second messenger, a role for zinc that has recently been suggested in immune cells 11, but rather through its role as a cofactor or as a structural element. The data presented here suggest that the zinc-dependent effect is upstream of the inflammasome and caspase-1 activation and acts by regulating pannexin-1 activity. The stimuli used to activate caspase-1 in this study do so *via* assembly of an NALP3 inflammasome, and it remains to be tested whether other stimuli for IL-1β release require a zinc-dependent mechanism. Currently we do not know whether pannexin-1 is the molecular partner for zinc in the model described here or whether another protein is involved in the mechanism. However, the findings presented here mean that this can now be investigated.

## Materials and methods

### Materials

RPMI 1640 and DMEM culture media, FBS, glutamine and a streptomycin/penicillin antibiotic solution were all purchased from Invitrogen. Bacterial LPS(*Escherichia coli* 026:B6), ATP, nigericin, ZnPyr (zinc salt), TTM, TPEN and pluronic acid were purchased from Sigma. SIH was purchased from ChemBridge (USA). The anti-mouse IL-1β antibody used for Western blot (S329) was a kind gift from the National Institute of Biological Standards and Controls (UK). The anti-mouse p10 caspase-1 antibody was from Santa Cruz Biotechnology. FluoZin-3 ester was purchased from Invitrogen.

### Cell culture

Macrophages were prepared from adult, male C57BL/6 mice (supplied by Harlan, UK), as described previously 15. Briefly, 24 h after isolation the macrophages were incubated with LPS (1 μg/mL, 2 h) to induce the expression of pro-IL-1β. The macrophages were then incubated with ATP (5 mM, 10 min) to induce caspase-1-dependent processing and release of IL-1β. HEK-293 cells stably expressing mouse P2X7-receptor were cultured in DMEM-F12 media supplemented with l-glutamine, 10% fetal calf serum and 300 μg/mL of G418 (all from Invitrogen).

### LDH assay

Release of the enzyme LDH from the cells was performed using the CytoTox-96 assay (Promega) according to the manufacturer's instructions.

### Western blotting

Supernatants and lysates were harvested and prepared in a sample buffer containing 1% β-mercaptoethanol. Samples were boiled and then electrophoresed on 12% (IL-1β) and 10% (caspase-1) SDS-acrylamide gels. Proteins were transferred to a nitrocellulose (IL-1β) or a polyvinylidene fluoride (PVDF) (caspase-1) membrane and blotted with primary antibodies, followed by HRP-conjugated secondary antibodies, and subsequent exposure using ECL reagents (Amersham, UK).

### Detection of IL-1β and IL-6 by ELISA

Measurement of IL-1β and IL-6 released into macrophage culture supernatants was done using specific mouse IL-1β and IL-6 ELISA kits (R&D Systems) following manufacturer's instructions.

### Caspase-1 assay

The activity of caspase-1 was measured after preparing a cell-free inflammasome extract as described in the *cell culture* section 19. Macrophages were primed with LPS for 2 h as described above. The cells (5×10^6^ cells/mL) were then lysed in a hypotonic lysis buffer (20 mM HEPES, pH 7.5, 1.5 mM MgCl_2_, 1 mM EDTA, 1 mM EGTA, 0.1 mM PMSF and protease inhibitor cocktail (Calbiochem)) for 15 min on ice. The resulting suspension was triturated with a pipette and then cleared of debris by centrifugation (10 min, 4°C, 13 000*g*). The supernatants were collected and incubated with 20 μM Ac-YVAD-AMC (Alexis Biochemicals) for 4 h at 4°C or at 37°C. Fluorescence was read using a BioTek Synergy HT multi-detection reader (excitation 380 nm, emission 460 nm).

### FluoZin-3 imaging

LPS-treated macrophages were incubated with 10 μM FluoZin-3 ester plus 0.02% pluronic acid for 40 min. The cells were then changed into RPMI 1640 containing 5% FBS, 100 μg/mL streptomycin, 100 IU penicillin and 20 mM HEPES, pH 7.3. All the imaging experiments were performed at room temperature. The imaging was performed using a BD Pathway Bioimager with liquid handling, exciting and collecting fluorescence emission with the Fluo 4 filter settings. The BD Pathway is part of the Core Bioimaging Facility at the Faculty of Life Sciences, University of Manchester (http://www.ls.manchester.ac.uk/research/facilities/bioimaging/). All offline analyses of images and movies used ImageJ software (http://rsb.info.gov/ij/).

In ImageJ, cells were selected as regions of interest and at least 15 cells *per* field of view could be analysed. The fluorescence (*F*) data are expressed as a fluorescence change relative to the initial base line fluorescence value (Fo).

### Fura-2 calcium assay

HEK-293 expressing mouse P2X7-receptor were plated in a 96-well black assay plate with clear bottom (Corning) and cultured overnight to reach 90–100% confluency. Cells were pre-incubated with 50 μM TPEN or vehicle (DMSO) and 4 μM Fura-2 AM (Molecular Probes) at 37°C for 30 min. Prior to recording, Fura-2 AM was removed and replaced with a standard extracellular solution consisting of 147 mMNaCl, 10 mM HEPES, 10 mM EGTA, 147 mM NaCl, 2 mM KCl, 1 mM MgCl_2_, 2 mM CaCl_2_, 10 mM HEPES and 13 mM glucose, pH 7.3, in the presence or absence of 50 μM TPEN. Fluorescence was recorded by an automatic fluorescence plate reader (FlexStation 3, Molecular Devices) over 120 s at 4 s intervals. The dual excitation for Fura-2 was 340 nm/360 nm and the emission was 510 nm. ATP (1 mM) was added into the wells automatically by the machine after 15 s of recording. The intracellular calcium level was expressed as the ratio of the emission intensities for 340 and 360 nm (F340/F360).

### Ethidium bromide uptake

Dye uptake experiments were performed as described previously 21,22. Briefly, peritoneal macrophages primed with 1 μg/mL of LPS for 2 h or HEK-293 expressing mouse P2X7-receptor plated on glass coverslips were incubated with TPEN (50 μM), ^10^panx1 mimetic peptide (WRQAAFVDSY, 400 μM, synthesised by AltaBioscience, UK) or vehicle (DMSO) for 20 min before recording. Ethidium bromide (20 μM, Sigma) was added 3 min before recording in standard physiological extracellular solution and fluorescent images were recorded on a Nikon confocal microscope under 20× objective at 4 s intervals for 100 s at 37°C, ATP (3 mM) was added after 24 s of recording. For each experiment, the time course of ethidium fluorescence was measured for 20 isolated cells and then averaged to obtain the mean fluorescent signal, the slope of the fluorescent signal *versus* the time was used as the most accurate and consistent measurement for comparisons.

### Data analysis

Data are presented as the mean±SD of at least three separate cultures. Groups of data were analysed by one-way ANOVA followed by Bonferronis multiple comparison test. Statistical significance was assumed when *p*<0.05. All Western blots and fluorescence profiles presented are representative of three independent experiments from three separate cultures.

## Abbreviations

HEK: human embryonic kidney
LDH: lactate dehydrogenase
NALP: NACHT-, LRR-, and PYD-containing protein
SIH: salicylaldehyde isonicotinoyl hydrazone
TPEN: N,N,N0,N0-tetrakis-(2-pyridylmethyl)ethylene diamine
TTM: ammonium tetrathiomolybdate
ZnPyr: 1-hydroxypyridine-2-thione
